# The m^6^A-related gene signature stratifies poor prognosis patients and characterizes immunosuppressive microenvironment in hepatocellular carcinoma

**DOI:** 10.3389/fimmu.2023.1227593

**Published:** 2023-08-25

**Authors:** Ensi Ma, Jianhua Li, Conghuan Shen, Yange Gu, Xinju Zhang, Li Li, Jing Zhao, Zhengxin Wang

**Affiliations:** ^1^ Liver Transplantation Center, General Surgery, Huashan Hospital, Fudan University, Shanghai, China; ^2^ Institute of Organ Transplantation, Fudan University, Shanghai, China; ^3^ Central Laboratory, Huashan Hospital, Fudan University, Shanghai, China; ^4^ Hepatobiliary Surgery, Department of General Surgery, Huashan Hospital & Cancer Metastasis Institute, Fudan University, Shanghai, China

**Keywords:** M 6 A modification, hepatocellular carcinoma, overall survival, cancer immunity, immunosuppressive microenvironment

## Abstract

**Background:**

N^6^-methyladenosine (m^6^A) is the most abundant epitranscriptomic modification of RNA, which can affect RNA metabolism and protein translation. The m^6^A modification plays a critical role in cancer development, including hepatocellular carcinoma (HCC). Despite several m^6^A-related signatures in HCC, most of them lack the necessary validation and the reliability is still elusive.

**Methods:**

Differentially expressed genes (DEGs) in the Cancer Genome Atlas were comprehensively analyzed to identify m^6^A signature associated with HCC prognosis. Gene set enrichment analysis, tumor mutation burden (TMB), immune infiltration, and therapeutic response were evaluated. Importantly, mass spectrometry proteomics and multiplex immunofluorescence assays were performed for validation.

**Results:**

The m^6^A-related protein-coding gene signature was established, which can divide HCC into high-/low-risk subgroups with markedly different overall survival (OS) and clinical stages. Furthermore, we validated its reliability and robustness in our 101 independent HCC specimens using proteomic detection and confirmed that our signature readily identified high-risk HCC patients with 3-year survival rates of 44.1% vs. 71.8% in the low-risk group. Functional analysis indicated that the high-risk group might stimulate the cell cycle and activate oncogenic pathways such as MAPK, mTOR, and VEGF, whereas the low-risk group mainly regulated amino acid, fatty acid, and drug metabolism. Additionally, the high-risk group had more TMB, upregulated immune checkpoint molecule expression, including PD-1, CTLA4, TIM3, and LAG3, and preferentially formed an immunosuppressive microenvironment. Accordingly, potential therapeutic responses showed that high-risk patients were potentially sensitive to inhibitors targeting the cell cycle and MAPK signaling, with patients possibly benefiting from immunotherapy. Moreover, multiplex immunofluorescence assays indicated that high-risk HCC samples displayed distinct immunosuppressive features, with abundant M2-polarized macrophages and T-regulatory cell infiltration.

**Conclusion:**

The m^6^A signature had a prominent capacity to evaluate OS and characterize the tumor immune microenvironment of HCC, which may serve as a useful approach for risk stratification management and provide a valuable clue to choosing rational therapeutic strategies.

## 1 Introduction

Globally, hepatocellular carcinoma (HCC) is the sixth most common malignancy and the fourth leading cause of cancer-associated death (1). Multiple regimens, including targeted-, chemo-, and immune-therapies, have been approved for HCC treatment (2, 3); however, overall prognosis and survival rates for patients with HCC remain dismal, with approximately 781,000 HCC deaths every year (1). Therefore, effective approaches must be developed to identify patients with poor prognoses. For high-risk HCC subgroups, better clinical management and rational treatment strategies are required to improve overall prognosis rates.

Currently, because of ongoing advancements in new cancer detection technologies, N^6^-methyladenosine (m^6^A) modifications of RNA molecules (4), which exert significant effects on RNA stability, export, splicing, or translation (5), have come to prominence. The m^6^A modification is catalyzed by an installed complex composed of multiple methyltransferases as “writers,” m^6^A RNA-binding proteins as “readers,” and demethylases as “erasers.” The well-established m^6^A writer methyltransferases include methyltransferase-like 3 (METTL3), METTL14, Wilms’ tumor 1-associating protein (WTAP), RNA-binding motif protein 15 (RBM 15), and zinc finger CCCH-type containing 13 (ZC3H13). These methyltransferases are responsible for adding methylated units to target RNA. m^6^A modification is a dynamically reversible process that can be removed by RNA demethylases, the fat mass and obesity-associated protein (FTO), and alkB homolog 5 (ALKBH5). The m^6^A-modified RNA can be recognized by various RNA-binding protein “readers” to determine RNA fate, including YTHDC1, YTHDF1/2/3, insulin­like growth factor mRNA­binding protein family (IGFBP1/2/3), and RBMX (6).

Accumulating evidence demonstrates that m^6^A modification has critical roles in malignant cancer evolution and is highly topical in the cancer biology field (7–12). Many studies have delineated how m^6^A modification regulates HCC development. For instance, the “writer” METTL14 mediates m^6^A-mediated EGFR methylation, thereby suppressing EGFR/PI3K/AKT activity and inhibiting epithelial–mesenchymal transition and metastasis in HCC (13). The m^6^A “reader” YTHDF1 promotes HCC cell autophagy by binding with m^6^A-modified ATG2A and ATG14 mRNAs to increase their translation rates under hypoxia (14). The “eraser” FTO is also involved in HCC progression and modulates cancer stem cell properties by demethylating SOX2, KLF4, and NANOG mRNAs (15). Given the important regulatory function of m^6^A modification, m^6^A-related genes may have promising molecular profiles for patient stratification. Recently, several m^6^A-related gene signatures were identified in different cancers, including HCC (16–20). However, most signatures lacked validation data in independent cohorts; thus, their reliability and robustness remain elusive.

In this study, we identified and validated an m^6^A-related protein-coding gene signature that could be used to indicate prognosis, characterize the tumor immune microenvironment, and predict potential treatment efficacy for patients with HCC. Our findings may provide risk stratification management and rational therapeutic strategies for high-risk HCC patients.

## 2 Materials and methods

### 2.1 Gene expression data from HCC samples

Raw gene expression data from HCC and the corresponding clinical information were extracted from the Cancer Genome Atlas (TCGA) database (https://portal.gdc.cancer.gov/). Transcript data from 374 tumor specimens and 50 normal specimens were included. HTseq counts were normalized based on the transcripts per million (TPM) method.

The exclusion criteria were as follows: (i) histologic diagnosis ruled out HCC; (ii) extremely low gene expression values; and (iii) incomplete clinical data and a follow-up time<30 days. ID conversion was conducted by Perl (Perl Programming Language version 5.30.1)

### 2.2 Construction of the m^6^A-related signature in HCC

To generate the m^6^A-related signature and quantify the m^6^A modification patterns of each patient, we aimed to construct a scoring system (called m6AScore) and assess all individuals with HCC.

Initially, we conducted consensus clustering analysis using the ConsensusClusterPlus package (21) in R statistical software (version 4.1.3, https://www.r-project.org/). Steps included data preparation with gene expression data from TCGA or other sources, applying preprocessing techniques, selecting the ConsensusClusterPlus algorithm, determining the optimal number of clusters using the scree plot, and interpreting cluster results through gene functional analysis, pathway enrichment, and correlation analysis with clinical features.

Then, the overlapped differentially expressed genes (DEGs) were obtained by Venn diagrams. Next, the prognostic value of protein-coding DEGs of the distinct m^6^A modification patterns was determined using Cox proportional hazards regression analysis. Moreover, the protein-coding DEGs with significant prognostic value were included in calculating the m6AScore. Given the prognostic protein-coding DEGs in HCC, principal component analysis (PCA) was then performed to establish the m6AScore. PCA is a dimension reduction method and has been extensively used in gene expression analysis (19, 22). Similar to a previous study, we added principal components (PC) 1 and 2 as the last gene signature scores.


$$m6AScore=\sum_{i}^{j}\left(PC1i+PC2i\right)$$

Where i and j are the order and the total number of m^6^A-related prognostic protein coding in HCC. Finally, the Z-score of the m6AScore was used for further analysis.

For different clusters, the outstanding gene ontology (GO) terms and Kyoto Encyclopedia of Genes and Genomes (KEGG) pathways were identified and visualized by bubble diagrams.

### 2.3 HCC participant enrollment

In total, an independent cohort of 101 patients with HCC was enrolled at Huashan Hospital, Fudan University, between March 2015, and June 2020. None of the patients received radiotherapy or chemotherapy before surgery. Each patient provided informed consent. The study protocol was approved by the Human Research Ethics Committee of Huashan Hospital, Fudan University. Basic information about the samples is listed in the **Supplementary Materials** (**Tables S4, S5**).

### 2.4 General mutation information

Copy number variation (CNV) data were downloaded from the TCGA database. Gene mutation data were also downloaded from the TCGA database, and gene mutations were identified in the high-/low-m6AScore groups. Moreover, the tumor mutation burden (TMB) of each sample was calculated via a Perl script (https://www.perl.org/). TMB was defined as the total number of mutations per megabase in the tumor tissue.

### 2.5 The gene set enrichment analysis (GSEA)

GSEA is a computational method that can execute GO and KEGG analyses with a given gene list. GSEA software version 4.2.3 (https://www.gsea-msigdb.org/) was employed to predict the potential functions of the m^6^A-related signature. Combined with the high- and low-risk groups determined by the m6AScore, GO and KEGG pathway enrichment analyses were conducted to visualize various genes involved in different pathways, biological functions, and their expression patterns. Data were corrected for multiple testing (number of permutations = 1000).

### 2.6 Prediction of the response to chemotherapy

The R package of pRRophetic was used to predict the sensibility of common chemotherapeutic agents, as previously described (23). Sensibility indicates the effectiveness of a substance in inhibiting specific biological or biochemical functions. The group difference was tested by the Wilcoxon signed-rank test.

### 2.7 Prediction of the response to immunotherapy

Gene signature analysis of T-cell dysfunction and prediction of cancer immunotherapy response by patients with cancer were performed using the tumor immune dysfunction and exclusion (TIDE) algorithm as previously described (24). TIDE utilized both T-cell dysfunction and exclusion signatures to model immune escape in tumors with different cytotoxic T lymphocyte levels, and the TIDE score is consistent with signatures of tumor immune evasion. A higher tumor TIDE score is associated with a worse immune checkpoint blockade response.

### 2.8 Immunocyte infiltration and immune function analysis

By performing single-sample gene set enrichment analysis (ssGSEA) (25), the immune function scores of each patient with HCC were calculated to further quantify the composition of tumor-infiltrating immune cells. According to the immune score, the degree of immune cell infiltration was quantified in HCC tissues. Immunocyte gene signatures enrolled in this study are listed in **Table S6**.

### 2.9 Mass spectrometry proteomic detection

Proteomic detection was performed in four steps: protein isolation from formalin-fixed paraffin-embedded tumor samples, protein digestion, peptide sequencing by MS, and data analysis to identify the proteins.

The details of the processing of the raw files generated by MS are as follows: MS raw files were searched in the NCBI Human Refseq database using the Mascot search engine (v2.3, Matrix Science Inc.; version 04-07-2013, total 32015 entries). The parent and daughter ion mass deviations were set at 20 and 50 ppm, respectively (QExactive HF) or 0.5 Da. The theoretical protein cleavage sites were arginine (R) and lysine (K), with a maximum of two missed cleavage sites allowed. Carbamidomethylation (C) for immobilization and acetyl (protein N-term) and oxidation (M) for dynamic modifications. All the identified peptides were obtained from the area under the MS1 peak calculation, and the peptide false discovery rate was controlled at 1%. At the protein characterization level, we only kept those proteins that contained at least one unique peptide and two high-quality peptides (strict peptide, i.e., Mascot Ion Score >20). For protein quantification, we used an intensity-based absolute quantification algorithm (i.e., iBAQ algorithm) and subsequently normalized each iBAQ value to FOT-iBAQ values. This was performed by dividing the iBAQ value by the sum of the iBAQ of all detected proteins in the corresponding sample. Then, to make this FOT size easier to read and write, this FOT value was multiplied by 105, resulting in the iFOT value.

The proteome sequencing of 101 HCC specimens obtained from the General Surgery Department of Huashan Hospital, Fudan University, was performed by the National Institute of Metrology (China) as described above. The sample quality control and the protein profile expression involved in this study have been shown in **Figures. S1, S2** in **Supplementary Material**.

### 2.10 Multiplex immunofluorescence assay

After the paraffin sections were dewaxed in water, the slides were immersed in citric acid antigen repair buffer and boiled in water for antigen repair. The sample was incubated with 3% H_2_O_2_ at room temperature before rinsing with phosphate-buffered saline (PBS). The solution was closed with 5% FBS at 37°C for 30 min and then incubated with the primary antibody at 4°C overnight. The sample was rinsed with PBS, incubated with Cy5/Sp Green-labeled secondary antibodies at 37°C, and then rinsed again with PBS. The solution was then incubated with Hoechst at room temperature for 15 min, and serial staining was performed repetitively by stripping off previous primary/secondary antibodies via microwave treatment.

### 2.11 Statistical analysis

The Kaplan–Meier analysis with the log-rank test was performed to compare the survival difference between the high-/low-risk HCC groups. Data were tested for normal distribution, and if normally distributed, the Student’s t-test was used or the nonparametric Mann–Whitney U-test was applied unless stated otherwise. Correlations were assessed according to the Pearson test for parametric data and the Spearman test for nonparametric data. All statistical analyses were conducted using Prism (version 8, https://www.graphpad-prism.cn/) and R statistical software (version 4.1.3, https://www.r-project.org/). A *P*-value of<0.05 denoted a statistically significant difference (*P< 0.05; **P< 0.01, and *** *P*< 0.001).

## 3 Results

### 3.1 Identification of the m^6^A-related gene signature in HCC

**Figure 1:**
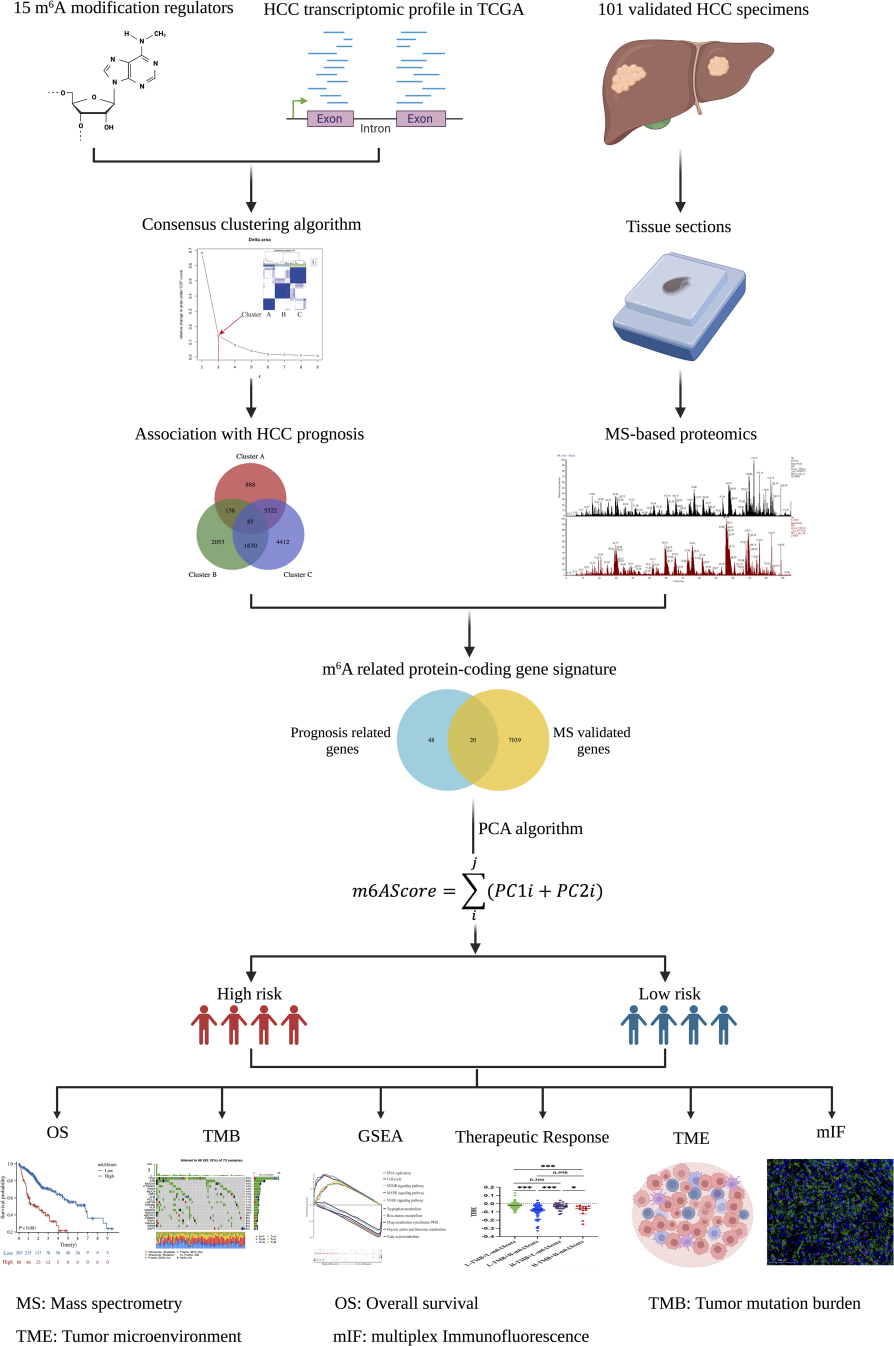
Flowchart of establishing the m^6^A-related protein-coding gene signature in HCC. The schematic diagrams were created with the help of BioRender.

The TCGA database, which includes detailed clinical information on 33 cancer types, is regarded as the landmark of cancer genomics programs. To identify the m^6^A-related signature in HCC, we downloaded all the raw gene expression data of 374 patients with HCC from the TCGA database. After carefully checking the clinical information of each sample, 31 patients were excluded because of incomplete clinical data or a follow-up time of less than 30 days. Ultimately, 343 patients with reliable transcript data and detailed clinical information were enrolled in the study. Through literature reports, 15 well-established m^6^A regulatory genes (*METTL3, METTL14, WTAP, ZC3H13, RBM15, YTHDC1, YTHDF1, YTHDF2, YTHDF3, IGFBP1, IGFBP2, IGFBP3, RBMX, FTO, and ALKBH5*) were used for further analysis. The whole analysis process was summarized as a flowchart in **Figure 1**.

**Figure 2:**
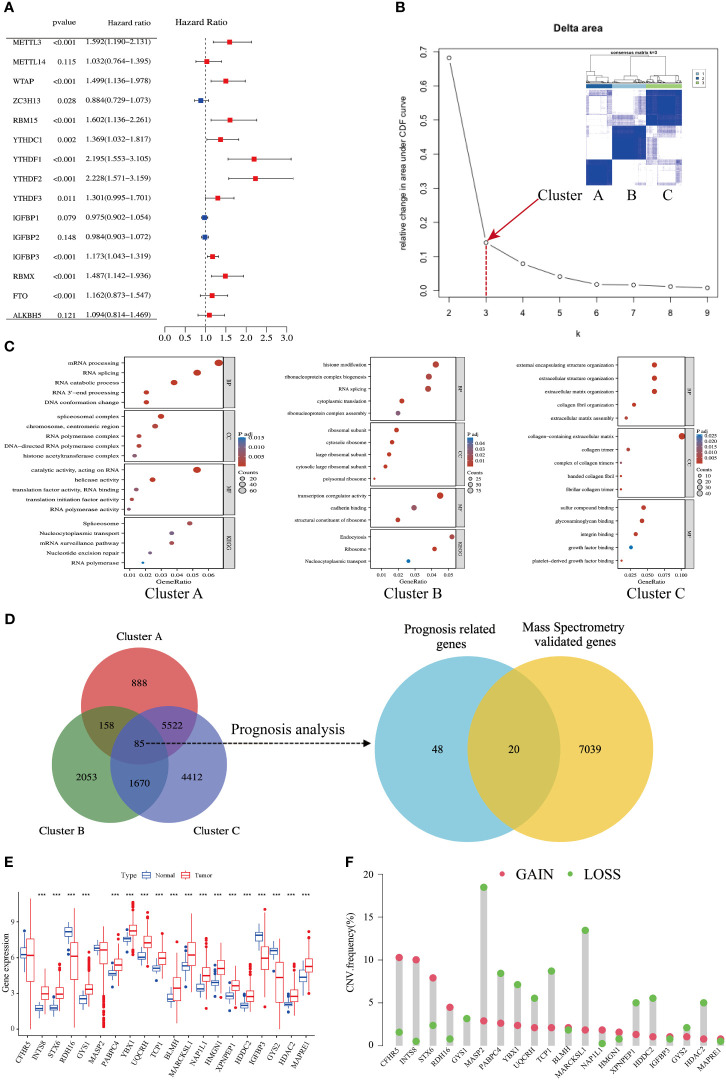
Identification of an m^6^A-related gene signature in HCC. **(A)** Forest plots show 15 m^6^A-related genes from uniCox regression analysis with respect to OS and gene expression. **(B)** A scree plot shows the optimal number of principal components; k = 3 was selected. **(C)** Bubble plots show GO and KEGG enrichment analyses of differentially expressed genes (DEGs) in three clusters. **(D)** Venn diagrams show DEGs in three clusters (left side); mass spectrometry-validated genes and prognosis-related genes (right side). **(E)** Expression levels of 20 genes in tumors and adjacent normal counterparts in HCC; red and blue boxes indicate tumor and normal tissue, respectively. **(F)** Copy number variation (CNV) frequency of the 20 genes in HCC; CNV gains and losses are shown in red and green, respectively.

Univariate Cox regression analysis of the gene expression level revealed that 11 of the 15 m^6^A regulators were significantly correlated with HCC prognosis, whereas *METTL14*, *IGFBP1/2*, and *ALKBH5* failed to reach statistical association (**Figure 2A**). According to the consensus algorithm with the 11 m^6^A regulators, the slope of the scree plot sharply declined when the abscissa was k = 3, suggesting that the patients could be divided into three categories for better cluster presentation (**Figure 2B**). GO and KEGG analyses showed that each cluster displayed different features (**Figure 2C**). Cluster A was closely associated with RNA processing and splicing (**Figure 2C**, left panel). Cluster B was involved in histone modification and ribosome assembly (**Figure 2C**, middle panel). Cluster C was mainly enriched in extracellular structure, collagen matrix, and integrin binding (**Figure 2C**, right panel). There were 85 DEGs overlapped in the three clusters (**Table S1**), and 68 of these DEGs were significantly associated with HCC prognosis (**Figure 2D**, left panel). DEGs are listed in **Table S2**. Given that protein-coding genes are major executors of biological function, we performed proteomic detection using MS assays on 101 HCC samples at our center. In total, 7059 protein expression were successfully detected using this approach. Subsequently, 68 prognosis-related genes were intersected with MS-detected genes, and 20 protein-coding genes were eventually defined as m^6^A-related signatures for analysis (**Figure 2D**, right panel). Their coefficients are listed in **Table S3**.

The expression levels of the 20 m^6^A-related genes were compared between HCC tissues and adjacent normal counterparts. Most of them were upregulated, and five of them were downregulated (**Figure 2E**). The copy number variation (CNV) of loss and gain is shown in **Figure 2F**. Moreover, we found an abundance of high-confidential m^6^A clusters within these 20 genes via SRAMP online analysis (http://www.cuilab.cn/sramp) (**Figure S3**), suggesting that these genes are m^6^A modification targets.

Taken together, we established an m^6^A-related signature composed of 20 protein-coding genes, which has a significant correlation with HCC prognosis.

### 3.2 Clinical significance of the m^6^A-related signature in HCC

**Figure 3:**
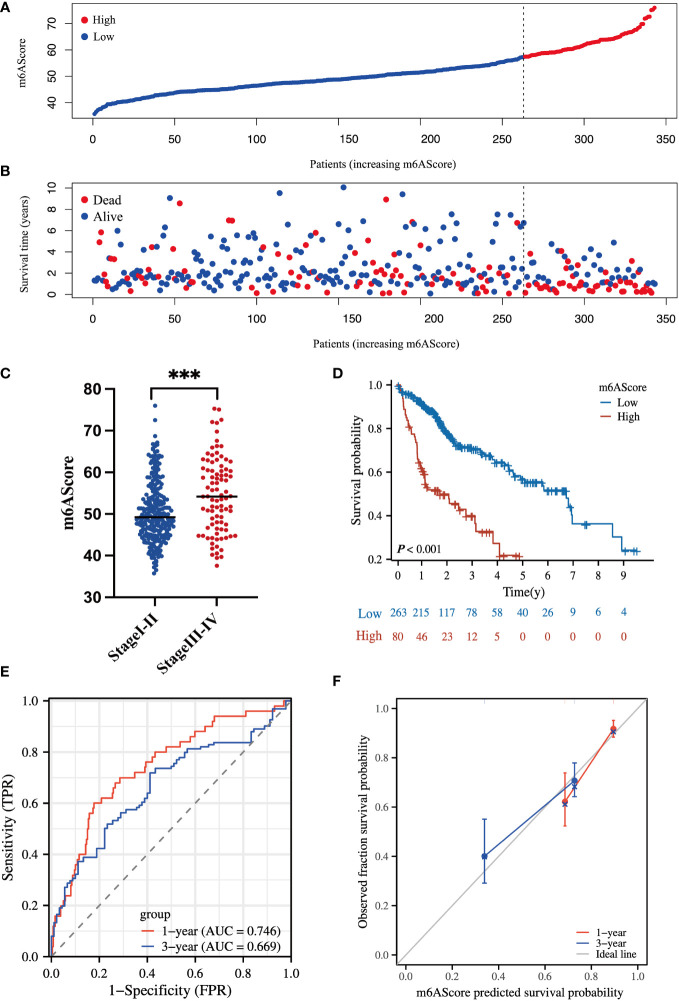
Clinical significance of the m^6^A-related signature in the TCGA-HCC cohort **(A)** m6AScore distribution according to the m^6^A-related signature in TCGA-HCC samples; red represents a high m6AScore (high risk) group and blue represents a low m6AScore (low risk) group. **(B)** Associations between the m6AScore and survival status; red represents deceased patients and blue represents surviving patients. **(C)** Box plot showing the relationship between the m6AScore and clinical pathological stage; blue represents stage I–II patients and red represents stage III–IV patients. **(D)** Kaplan–Meier survival curves showing the low m6AScore group (blue graph) and the high m6AScore group (red graph). **(E)** Receiver operating characteristics (ROC) curve and area under the ROC of m6AScores for 1 (red graph) and 3 (blue graph) years. **(F)** Calibration curve of m6AScores for 1 (red graph) and 3 (blue graph) years.

According to the m^6^A-related signature, the m6AScore was calculated for each patient, as described in the method. Based on the Youden index (26), 343 HCC patients in TCGA were divided into low- and high-risk groups. As shown in **Figures 3A, B**. the death risk gradually increased with the elevation of m6AScore. Meanwhile, a high m6AScore was markedly correlated with advanced HCC stages (**Figure 3C**). Thus, patients with high m6AScores were stratified into the high-risk HCC group.

Then, Kaplan–Meier analysis for overall survival (OS) showed that the high-risk group frequently had a shorter survival time (**Figure 3D**). The 1-, 3-, and 5-year survival rates of high-risk vs. low-risk patients were 62.2% vs. 91.7%, 40.1% vs. 70.7%, and 21.9% vs. 57.0%, respectively. Receiver operating characteristic (ROC) curve analysis indicated that the m6AScore was a useful prognostic index for OS prediction of patients with HCC, as the areas under the ROC curve (AUCs) reached 0.746 and 0.669 in 1 and 3 years, respectively (**Figure 3E**). Moreover, the m6AScore model displayed good calibration, and the calibration curves for 1- and 3-year survival were all close to the ideal line (**Figure 3F**).

To further confirm the reliability of our model, we conducted validation using an external dataset of ICGC-JP. As shown in **Figure S4**, the 1-, 3-, and 5-year survival rates of high-risk vs. low-risk patients were 84.8% vs. 94.9%, 71.2% vs. 84.5%, and 40.7% vs. 70.0%, respectively. The 1- and 3-year AUC was 0.707 and 0.739. These results indicated that our model had a favorable predictive activity.

Collectively, these findings suggest that the m^6^A-related coding-gene signature has a great capacity to predict the OS of patients with HCC.

### 3.3 Proteomic validation of the m^6^A-related gene signature in an independent HCC cohort

To evaluate the reliability of the m^6^A-related protein-coding gene signature in prognostic evaluation, an independent cohort of 101 HCC specimens was used for further validation. The basic information about the validated HCC cohort in our center is listed in **Table S4**. The protein levels of m^6^A-related signature were determined by MS assay, and the m6AScore was calculated to divide patients into high- and low-risk HCC groups as above mentioned.

**Figure 4:**
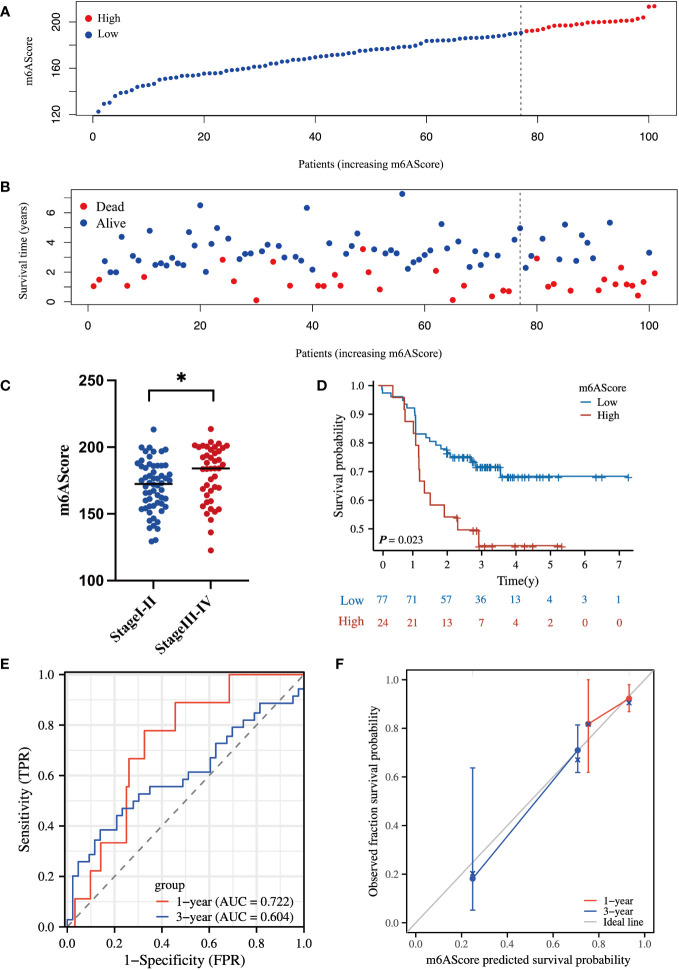
Proteomic validation of the m^6^A-related gene signature in an independent HCC cohort **(A)** m6AScore distribution according to the m6A-related signature in independent HCC samples; red represents a high m6AScore (high risk) group and blue represents a low m6AScore (low risk) group. **(B)** Associations between the m6AScore and survival status; red represents deceased patients and blue represents surviving patients. **(C)** Box plot showing the relationship between the m6AScore and clinical pathological stage; blue represents stage I–II patients and red represents stage III–IV patients. **(D)** Kaplan–Meier survival curves showing the low m6AScore group (blue graph) and the high m6AScore group (red graph). **(E)** Receiver operating characteristics (ROC) curve and area under the ROC of m6AScores for 1 (red graph) and 3 (blue graph) years. **(F)** Calibration curve of m6AScores for 1 (red graph) and 3 (blue graph) years.

In line with the above results in **Figure 3**, the high m6AScore group had a higher death risk (**Figures 4A, B**) and advanced clinical stages (**Figure 4C**). In addition, the high-risk group had a remarkably shorter survival time (**Figure 4D**). The 1-, 3-, and 5-year survival rates of high-risk vs. low-risk patients were 87.5% vs. 92.2%, 44.1% vs. 71.8%, and 44.1% vs. 68.3%, respectively. ROC curve analysis further confirmed the accuracy and sensitivity of the m^6^A-related signature, as the AUC reached 0.722 and 0.604 in 1 and 3 years, respectively (**Figure 4E**). The calibration curves for 1 and 3 years were close to the ideal line and showed a satisfactory calibration effect (**Figure 4F**).

Therefore, these validated results confirm the reliability and robustness of our m^6^A-related signature that can readily stratify high-risk HCC patients and evaluate OS. The m^6^A-related signature may facilitate better clinical management of high-risk HCC at an early stage.

### 3.4 Functional characterization of the m^6^A-related gene signature and potential therapeutic strategy for high-risk HCC groups

**Figure 5:**
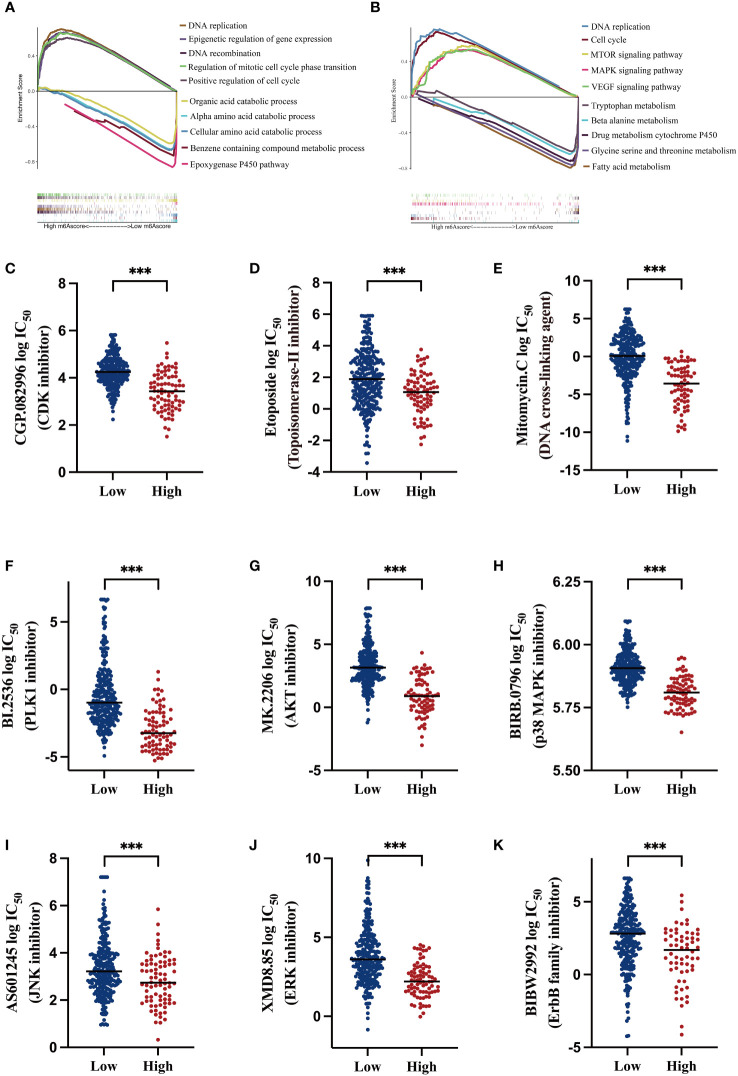
Functional analysis of the m^6^A-related signature and predicting susceptibility to targeted/chemotherapy agents **(A)** Gene Set Enrichment Analysis (GSEA) of gene ontology (GO) terms; the lower section shows gene set enrichment in the low m6AScore group and the upper section shows gene set enrichment in the high m6AScore group. **(B)** GSEA for KEGG pathways; the lower section shows gene set enrichment in the low m6AScore group and the upper section shows gene set enrichment in the high m6AScore group. **(C–K)** Box plots showing estimated logIC_50_ values for several chemotherapeutic agents in high- or low-m6AScore groups. *** represents p < 0.001.

To exploit the underlying mechanisms of the m^6^A-related signature, GSEA was performed for GO and KEGG analyses to explore possible biological functions. As shown in **Figures 5A, B**, in the high-risk HCC group, aberrant DNA replication and recombination, epigenetic dysregulation, cell cycle chaos, and activation of oncogenic signaling pathways such as MAPK, mTOR, and VEGF were obviously enriched. In contrast, disordered amino acid and fatty acid metabolism as well as abnormalities of the cytochrome p450 pathway were markedly enriched in the low-risk HCC group.

Based on GSEA results, we probed potentially effective strategies for high-risk HCC treatments. Some Food and Drug Administration-approved inhibitors and chemotherapeutics that target the cell cycle (CGP.082996), repress DNA replication and synthesis (etoposide and mitomycin C), and inactivate MAPK signaling (PLK1, AKT, and p38 inhibitors) were selected for analysis. Then, half maximal inhibitory concentrations (IC_50_) for these inhibitors were evaluated in low- and high-risk subgroups in the pRRophetic platform, which is an independent and authorized public resource for predicting inhibitor sensitivity and is based on gene expression microarray data (23). As shown in **Figures 5C–K**, high-risk HCC patients were more sensitive to these inhibitors, as low drug concentrations effectively inhibited cell proliferation.

These findings suggest that low- and high-risk groups may have different oncogenic mechanisms promoting HCC development. High-risk HCC patients may have elicited better responses to CDK and MAPK inhibitors and the chemotherapeutics etoposide and mitomycin C. Thus, our m^6^A-related signature was beneficial in selecting rational therapeutic strategies.

### 3.5 The m^6^A-related signature is associated with tumor mutation burden and immune checkpoint blockade therapy

**Figure 6:**
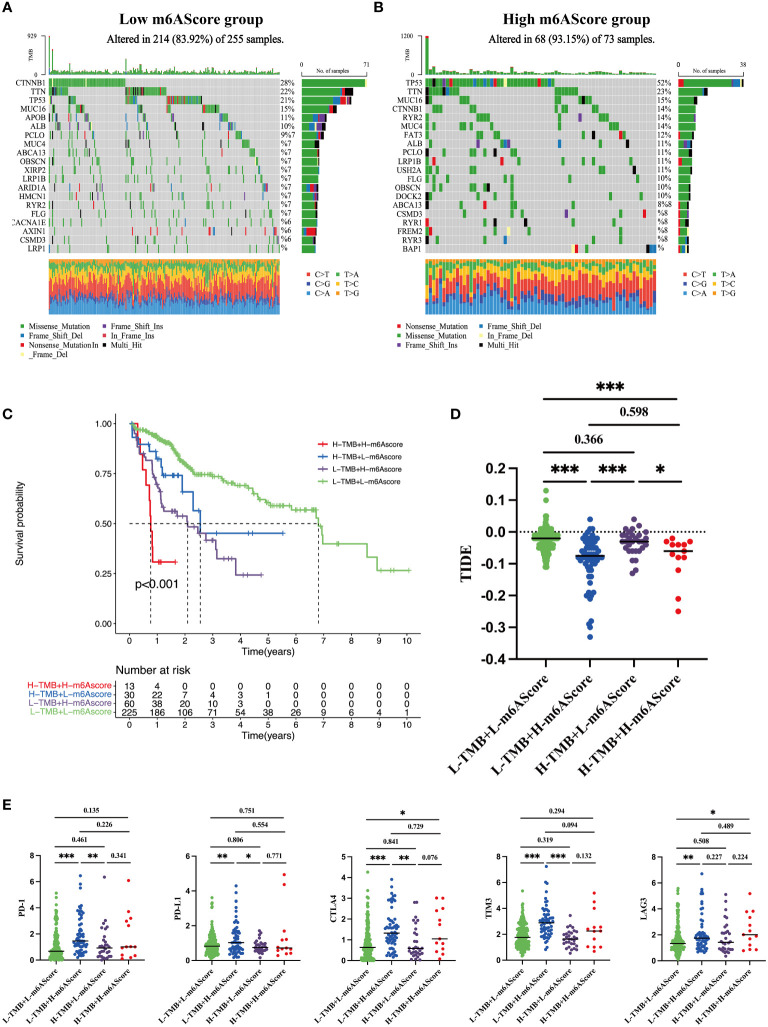
Tumor somatic mutations in the m^6^A-related signature and immunotherapy sensitivity predictions **(A)** Gene mutation frequency in the low m6AScore group. **(B)** Gene mutation frequency in the high m6AScore group. **(C)** Kaplan–Meier survival curves among four groups divided by tumor mutation burden (TMB) and m6AScores. L-TMB was the short for low TMB and H-TMB referred to high TMB; L-m6AScore was the short for low m6AScore and H-m6AScore referred to high-m6AScore. **(D)** TIDE analyses predict responses to immunotherapy as indicated by TIDE scores in the four groups; lower TIDE scores suggest increased responsiveness to immunotherapy. **(E)** Box plots show differential immune checkpoint gene expression (PD-1, PD-L1, CTLA4, TIM3, and LAG3) between high- and low-m6AScore groups. *, **, *** represent p < 0.05, p < 0.01, p < 0.001.

TMB is an important index to evaluate cancer progression and the efficacy of immunotherapy. We found that the gene mutation landscapes were markedly different between the low- and high-risk groups, as shown in the waterfall chart in **Figures 6A, B**. Among the low-risk patients, the CTNNB1 mutation was the most common event (**Figure 6A**), whereas in high-risk group, the frequency of the TP53 mutation appeared in more than half of the total mutant events (**Figure 6B**). Then, according to the combination of high/low TMB and m6AScore, HCC patients were divided into four subgroups as indicated in **Figure 6C**, L-TMB+L-m6AScore, L-TMB+H-m6AScore, H-TMB+L-m6AScore, and H-TMB+H-m6AScore. We found that the H-TMB+H-m6AScore group had the worst prognosis, whereas the L-TMB+L-m6AScore group had the best prognosis.

Recently, immunotherapy combined with immune checkpoint inhibitor approaches has generated encouraging clinical outcomes in different cancers, including HCC (27). TIDE is an algorithm that predicts immunotherapy responses; a high TIDE score frequently indicates poor responses (24). HCC patients with high m6AScores were inclined to have lower TIDE scores regardless of the TMB (**Figure 6D**), suggesting that high-risk groups benefit from immunotherapy. Furthermore, we analyzed the expression levels of several well-documented immune checkpoint molecules, including CTLA4, PD-1, PD-L1, LAG3, and TIM3, in TMB + m6AScore subgroups. Most immune checkpoint molecules had higher expression levels in the high m6AScore subgroup, indicating that high-risk HCC patients may suppress T-cell activation and evade antitumor immunity (**Figure 6E**). However, it also indicated that high-risk HCC patients may be sensitive to immune checkpoint therapy, such as antibodies directed against PD-1, CTLA4, TIM3, and LAG3.

Taken together, m6AScores combined with TMB could be used to stratify the poorest prognostic HCC subgroup. High-risk HCC patients are prone to immune evasion by upregulating immune checkpoint protein expression and may benefit from immune checkpoint therapy.

### 3.6 The m^6^A signature can characterize the tumor immune microenvironment

**Figure 7:**
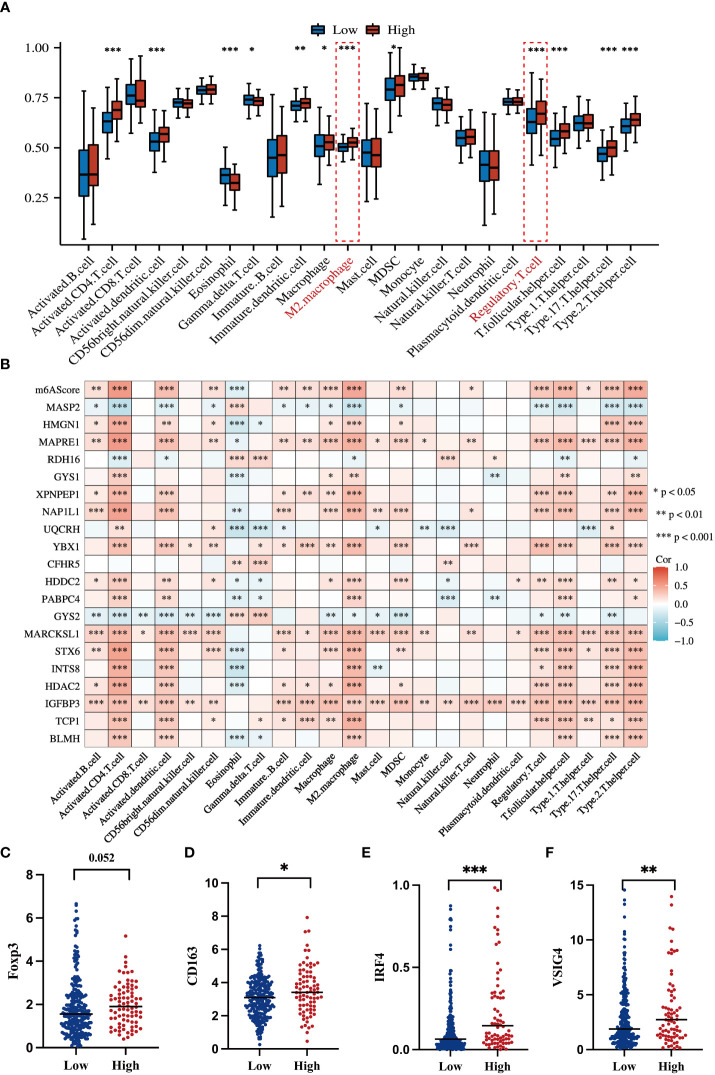
Correlations of the m^6^A-related signature with immune cell infiltration and related genes **(A)** Box plot shows an ssGSEA analysis of m6AScores and immune infiltration levels in HCC; red represents the high m6AScore group and blue represents the low m6AScore group. **(B)** Correlation heatmap showing the relationship between the m6AScore, gene expression (20 genes), and immune cell infiltration. **(C–F)** Box plots showing T-regulatory cell and M2-type macrophage-related gene expression between high- and low-m6AScore groups. *, **, *** represent p < 0.05, p < 0.01, p < 0.001.

To systematically delineate the status of the tumor immune microenvironment, we compared the infiltration percentage of various immune cells via ssGSEA analysis. Notably, immunosuppressive cells such as M2 polarized macrophages and T-regulatory cell were obviously accumulated in the high-risk group (**Figure 7A**). Each of the 20 genes in the m^6^A signature individually contributed to immune cell infiltration (**Figure 7B**). It is well known that Foxp3 is an essential transcriptional factor of Treg; CD163, IRF4, and VSIG4 are the markers of M2-macrophages. Thus, the expression levels of these genes were analyzed in the TCGA dataset. The high-risk HCC group exhibited significant upregulation of *Foxp3*, *CD163*, *IRF4*, and *VSIG4* (**Figures 7C–F**).

**Figure 8:**
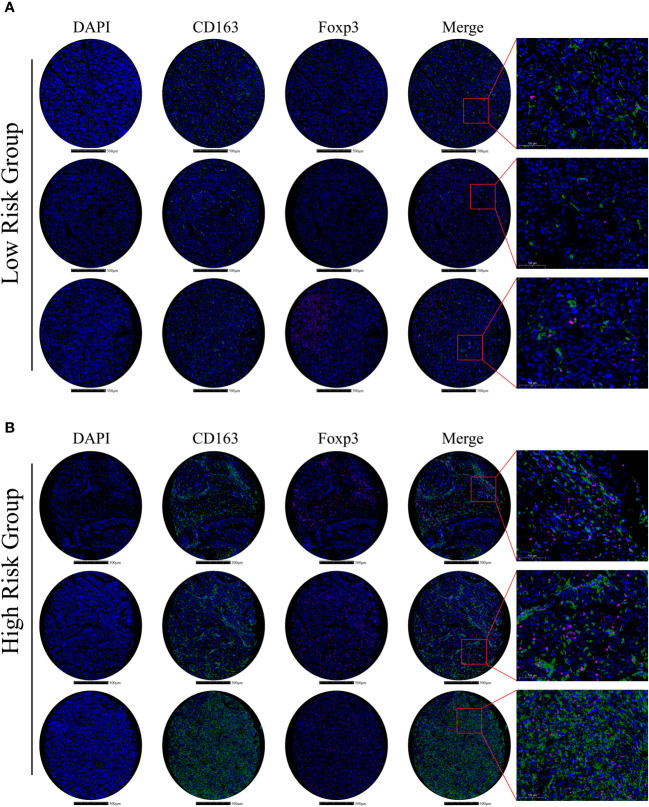
Immune-related gene expression and validation in HCC samples **(A)** In the low m6Ascore group, multiplex immunofluorescence staining was performed using DAPI, CD163, and Foxp3 stains; stains are shown separately and merged (Scale bars are shown in figures). **(B)** In the high m6Ascore group, the same multiplex immunofluorescence staining was conducted; stains are shown separately and merged (Scale bars are shown in figures).

To confirm and validate these findings, multiplex immunofluorescence staining was performed to display tumor microenvironment features in part of our HCC cohort. In low-risk HCC samples, there were a few M2-macrophages (green) and Tregs (purple) observed (**Figure 8A**). Consistently, in high-risk HCC samples, there was obvious infiltration of M2-macrophages and Tregs within the tumor microenvironment (**Figure 8B**).

Hence, these results indicate that m^6^A signature can characterize the tumor immune microenvironment and that the high-risk HCC group may form an immunosuppressive microenvironment with plenty of M2-macrophages and Treg infiltration.

## 4 Discussion

Globally, HCC is a heavy health burden on society due to its high morbidity and mortality rates (1). Thus far, effective early diagnosis and prognostic evaluation strategies are lacking for the disease. HCC has highly heterogeneous characteristics, manifested by highly frequent genomic variation, aberrant epigenetic modification, and dysregulated transcriptomic expression (28). Currently, epi-transcriptomic modification, represented by m^6^A RNA methylation, has been shown to have essential roles in cancer development, including HCC (29). Several genes are m^6^A modification targets and help mediate tumorigenesis, metastasis, and drug resistance (30). Herein, we propose that m^6^A-related gene signatures have great potential as promising biomarkers and therapeutic targets. Recently, several m^6^A gene profiles were reportedly associated with HCC progression (16–18, 20). For instance, Zheng et al. constructed a signature comprising only three m^6^A regulators, namely YTHDF2, METTL14, and ZC3H13 (17). Shen et al. identified an m^6^A methylation panel that clustered HCC into three distinct metabolic subtypes (16). Furthermore, m^6^A- and ferroptosis-related long non-coding RNA profiles were identified in HCC (18). However, most signatures lack validation in independent cohorts; therefore, their reliability and accuracy remain unclear. In this study, we mainly focused on protein-coding genes as they are the essential executors of biological function. We established an m^6^A-related signature containing 20 protein-coding genes by analyzing TCGA-HCC transcriptomic data and MS-proteomic-based detection in HCC samples. This way, we stratified high-risk HCC patients and characterized the immunosuppressive tumor microenvironment. Moreover, we independently validated our m^6^A-related gene panel using proteomic analyses in 101 HCC samples from our center. We confirmed that the high m6AScore HCC subgroup often had shorter survival times. Furthermore, in multiplex immunofluorescence assays, we delineated tumor microenvironment features between low- and high-m6AScore samples and identified more Treg and M2 macrophage infiltration in high-risk samples. This successful validation suggested that our m^6^A-related gene signature was reliable and robust in evaluating HCC prognosis rates.

To preliminarily understand the m^6^A-related gene signature, the m^6^A status of 20 protein-coding genes was analyzed in the SRAMP database. Most genes contained high-confidential m^6^A clusters, suggesting that they functioned as potential m^6^A modification targets (**Figure S3**). Indeed, several genes in the m^6^A-related signature, such as *HDAC2*, *GYS2*, and *IGFBP3*, were previously shown to regulate cancer development in an m^6^A-dependent manner; IGFBP3 potentially functions as a downstream effector of the YTHDF2-mediated the m^6^A-MYC axis to promote glioblastoma stem cell growth (31). GYS2 mRNA was m^6^A modified by METTL3 and recognized by IGFBP2 to stabilize mRNA transcription, thereby controlling hepatic glycogenesis (32). HDAC2 downregulated expression of the m^6^A reader, YTHDF2, by modulating clear cell renal cell carcinoma sensitivity to sunitinib therapy (33). Additionally, our GO, KEGG, TMB, and ssGSEA immune cell infiltration analyses identified underlying mechanisms leading to high-risk HCC outcomes. Aberrant MAPK, mTOR, and VEGFA signaling activation and a high TMB potentially account for malignant progression in high-risk HCC patients (**Figures 4B**, **5B**). With respect to the tumor immune microenvironment, the high-risk group tended to form an immunosuppressive microenvironment characterized by distinct Treg and M2 macrophage infiltration (**Figures 7**, **8**). Treg cells can hinder protective immunosurveillance and hamper effective immune responses to tumors, thus accelerating cancer progression (34). M2 macrophages share characteristics with tumor-associated macrophages (TAMs), which promote tumor initiation, metastasis, and malignancy (35). Previous studies reported that dysregulated m^6^A modification modulated Treg cell and TAM infiltration in tumors (36, 37). Thus, the tumor immunosuppressive microenvironment induced by Treg and M2 macrophages may be another important factor leading to a poor prognosis in high-risk HCC patients.

Given malignant tumor stages and shorter OS rates in the high-risk group, we explored potential treatment strategies. First, using oncogenic signaling pathway enrichment in high-risk patients, potential inhibitor sensitivity toward these pathways was analyzed. High-risk patients may benefit from AKT, MAPK, JNK, and ERK inhibitor treatments, as these agents kill HCC cells at relatively low drug concentrations (**Figures 5C–K**). In previous decades, immune checkpoint inhibitors have revolutionized HCC treatment. Combined atezolizumab (anti-PD-L1) and bevacizumab (anti-angiogenesis) showed significant improvements in prolonging OS rates when compared with traditional first-line HCC treatments with sorafenib or lenvatinib (38). TMB and positive immune checkpoint molecule expression are both commonly used as biomarkers in selecting responsive patients (39). We found that *CTLA4*, *PD-1*, *LAG3*, and *TIM3* were upregulated in high m6AScore HCC samples (**Figure 6E**). High-risk HCC patients may benefit from immunotherapy based on our TIDE analysis (**Figure 6D**). Accordingly, inhibitors targeting MAPK signaling, combined with immune checkpoint blockade, may be a promising strategy for high-risk HCC patients. Certainly, this approach requires comprehensive validation in future studies; however, our findings suggest that our m^6^A-related signature provides a rational therapeutic strategy for HCC patients.

Apart from the important clinical implications of our m^6^A-related signature, our study had some limitations. First, although some high-confidence m^6^A clusters were predicted for these genes, whether these 20 protein-coding genes are modified by m^6^A or whether they exert effects on m^6^A methylation remains to be clarified. Secondly, it is unclear how these genes cooperatively regulate HCC progression via m^6^A-associated mechanisms. Additionally, model reliability must be evaluated in future studies, and drug sensitivity validation in high-risk groups must be conducted in prospective, large HCC study cohorts.

In summary, we established an m^6^A-related protein-coding gene signature to stratify high-risk HCC patients. Moreover, we validated these findings in an independent HCC cohort by MS proteomic detection and characterized the tumor immune microenvironment by multiplex immunofluorescence assay. The model showed prominent potential to evaluate HCC survival and immunosuppressive status. Our m^6^A signature provides an alternative strategy for risk stratification and better clinical management of patients with HCC.

## Data availability statement

The data presented in the study are deposited in the TCGA repository, ICGC Data Portal, and supplementary material.The TCGA accession number: phs000178 (https://portal.gdc.cancer.gov/projects/TCGA-LIHC), and the ICGC Data Portal accession number: EGAS00001001692 (https://dcc.icgc.org/releases/current/Projects/LIRI-JP).

## Ethics statement

The studies involving human participants were reviewed and approved by Ethics Committee of Huashan Hospital Affiliated to Fudan University. The patients/participants provided their written informed consent to participate in this study.

## Author contributions

JZ and ZW conceived and designed the study. EM, JL, CS, XZ, YG, and LL collected data, constructed the model, and collected hepatocellular carcinoma samples. EM, JL, and CS performed the experiments. JZ and ZW analyzed the data and drafted the manuscript. All authors critically revised the work and approved the final manuscript.
