# Supplementary material for: The m6A-related gene signature stratifies poor prognosis patients and characterizes immunosuppressive microenvironment in hepatocellular carcinoma
Source: Front Immunol. 2023 Aug 25;14:1227593. doi: 10.3389/fimmu.2023.1227593 (PMC10485364; doi:10.3389/fimmu.2023.1227593)
Supplement: Supplementary file 1 [file DataSheet_1.pdf]

The mass spectra BasePeak spectra of part of the samples in the project are shown in Figure S1.

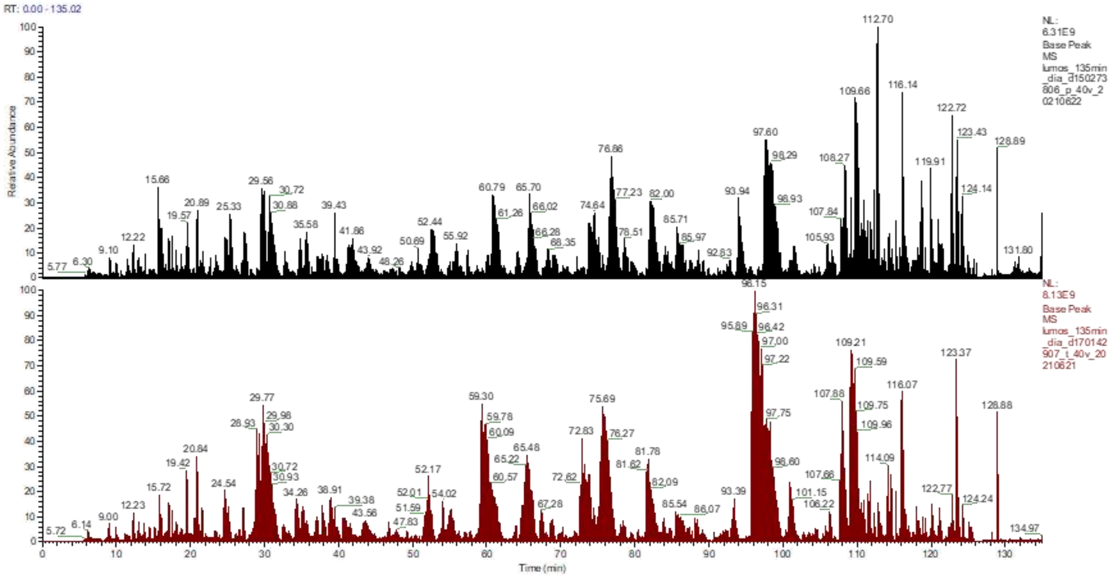

Figure S1. D15 and D17 mass spectra Basepeak Spectrogram

The results of protein identification for each sample are shown in Figure S2. The amount of protein identification for each sample was more than 4000, and most of the samples were in the range of 4500-5000.

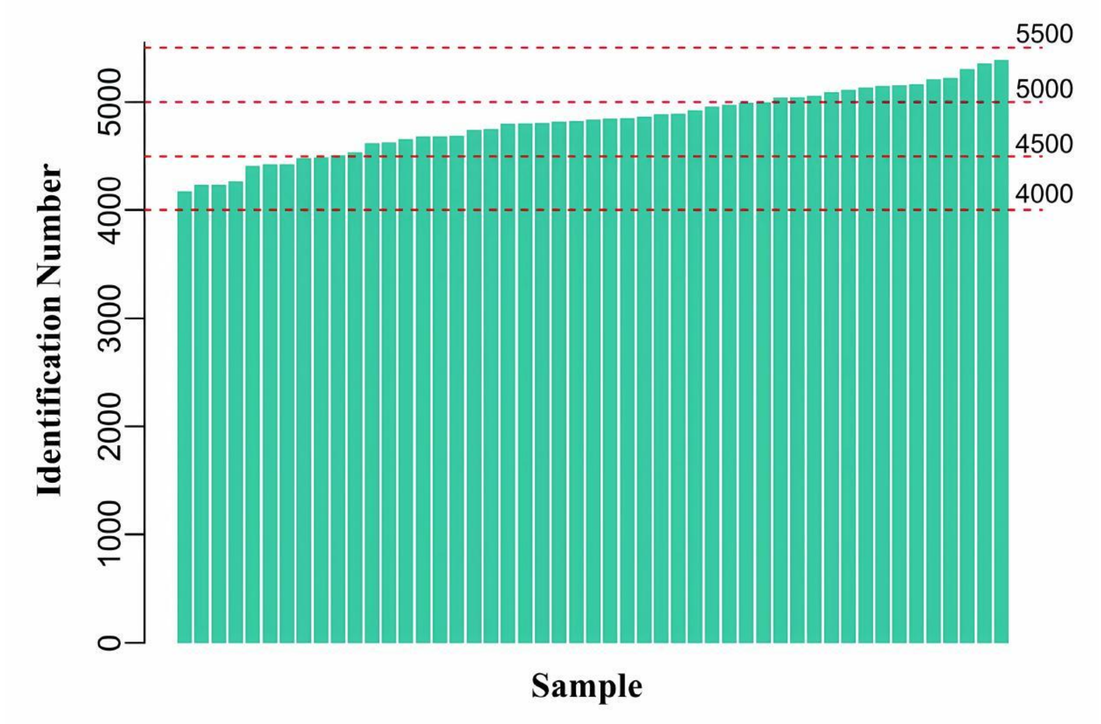

Figure S2. Number of protein identifications of FFPE tissue samples

1. Project experiment process

The main steps of FFPE sample proteome research process include: establishment of FFPE (Formalized fixed scaffold embedding) sample protein digestion scheme, LC-MS/MS analysis, establishment of spectrum library, database retrieval, data analysis, etc

2. Experimental process and method

2.1 Sample information

Sample quantity: 101 cases

2.2 Experimental reagents and instruments

1) Ammonium bicarbonate (Sigma, A6141)

2) Acetonitrile (Millipore, 1.00030.4008)

3）Trypsin （Promeg，V5113）

4) Centrifuge (Thermo Scientific, sorvall legal Micro 17)

5) Merck, Milli-Q Type 1 Ultra Water Systems

6) Mass spectrometer (Thermo Scientific, Orbitrap Fusion Lumos Tribrid)

7) Chromatographic system (Thermo Scientific, Easy-nLC1200)

8) Trap column, 100μm*20mm (3um, C18))

9) Chromatographic column (150μm*300mm (1.9um, C18))

2.3 Orthogonal experiment to determine FFPE sample digestion experiment scheme

In this project, orthogonal experiments were designed from paraffin removal, protein extraction and protein digestion to determine the optimal experimental scheme for FFPE samples.At present, the best paraffin removal schemes include Xylene and Triton X-100, protein extraction schemes include TFE and RapiGest, and protein digestion schemes include SP3 and IST.There are eight combination schemes in the orthogonal experiment, which are named TTI, TTS, TRI, TRS, XTI, XTS, XRI, XRS, respectively. Through proteome analysis of the same sample, the optimal experimental scheme is screened.

2.4 Construction of mass spectrometry reference library for early hepatocellular carcinoma

In this study, the proteome mass spectrometry data reference library was constructed from fresh hepatocellular carcinoma and adjacent tissues.The tissue protein was digested by FASP (Filter sided sample preparation) digestion scheme, and the peptide was divided into 10 fractions by C18 Tip for mass spectrometry analysis.Through MaxQuant and Spectraut analysis, we established the reference database of proteome mass spectrometry of early hepatocellular carcinoma, which contains 115346 parent ions, 97046 modified peptides, 690249 fragment ions, 89477 characteristic peptides, 85262 protein characteristic peptides, and 8798 protein identifications.

2.5 FASP digestion scheme

500μg for each sample Add protein and add 8M UA to dilute to 500μl. Transfer to 30 kd ultrafiltration tube and centrifugate at 14000 g for 20 min;After removing the waste liquid, add 200μL for each 8M UA, centrifugation at 14000 g for 15 min;After removing the waste liquid, add 100μL 20 mM DTT to each sample, shake for 1 min, and put it in 37 ℃ incubator for reaction for 4 h; After protein reduction, 14000 g centrifugation for 15 min to remove the waste liquid;Then carry out alkylation reaction, and add 100μL 50 mM IAA to each sample, vortex oscillation for 1 min, reaction in dark place at room temperature for 30 min, centrifugation to remove waste liquid after reaction;Then wash the ultrafiltration tube with 8 M UA and ammonium bicarbonate solution in turn;After washing, Trypsin enzyme was added for digestion for 16h. Finally, the peptides were collected by centrifugation.

2.6 DDA Mass Spectrometry Collection Method Settings

Setting of mass spectrum acquisition conditions: fragmentation mode is High energy collision dissociation (HCD), ionization mode is Nano ESI, and scanning is conducted in positive ion mode;Primary mass spectrum parameters: the range of ion scanning is 300~1550 m/z, the resolution is 120000, the automatic gain control (AGC) is $4e10^5$, and the maximum ion implantation time is 50 ms;Secondary mass spectrometry, resolution 15000, AGC $5e10^4$, maximum ion implantation time 30 ms,

quality data collected by XCalibur in real time.

2.7 DIA mass spectrometry analysis method

The variable isolation window DIA method with 30 windows and 40 windows was developed for mass spectrometry acquisition.The variable window is designed based on the spectral library.According to the m/z distribution of precursor ions in the spectral library, the number of precursor ions is equally distributed in each window.The final optimization method includes a full scan and 40 variable window DIA scans.The full scan resolution is set to 60000, the scan range is 350-1500 m/z, and the maximum ion implantation time is 50 ms;The DIA scanning resolution is set to 30000, the scanning range is 200-2000, the AGC is set to Standard mode, the maximum ion implantation time is 54 ms, and the cycle time is 3 s.

2.8 Database retrieval

The mass spectrum database retrieval software used in this project is MaxQuant 1.6.12.0 and Spectraut 14.9; The following protein database was used: 20375 protein sequences from Uniprot, species Human, downloaded on September 11, 2020.The MaxQuant database search software analysis parameter settings are shown in the figure below.

MaxQuant Analysis Parameter Settings

Item	Value
Enzyme	Trypsin
Max Missed Cleavages	2
Precursor Tolerance （Main search）	4.5 pm
Precursor Tolerance （First search）	20 ppm
MS/MS Tolerance	20 ppm
Fixed modifications	Carbamidomethyl (C)
Variable modifications	Oxidation (M) , Acetyl (Protein N-term)
Database	uniprot-human-20375-20200911
PSM FDR	0.01
Protein FDR	0.01

Min score for unmodified peptieds	20
Min score for modified peptides	40
