# Supplementary material for: The m6A-related gene signature stratifies poor prognosis patients and characterizes immunosuppressive microenvironment in hepatocellular carcinoma
Source: Front Immunol. 2023 Aug 25;14:1227593. doi: 10.3389/fimmu.2023.1227593 (PMC10485364; doi:10.3389/fimmu.2023.1227593)
Supplement: Supplementary file 2 [file DataSheet_2.pdf]

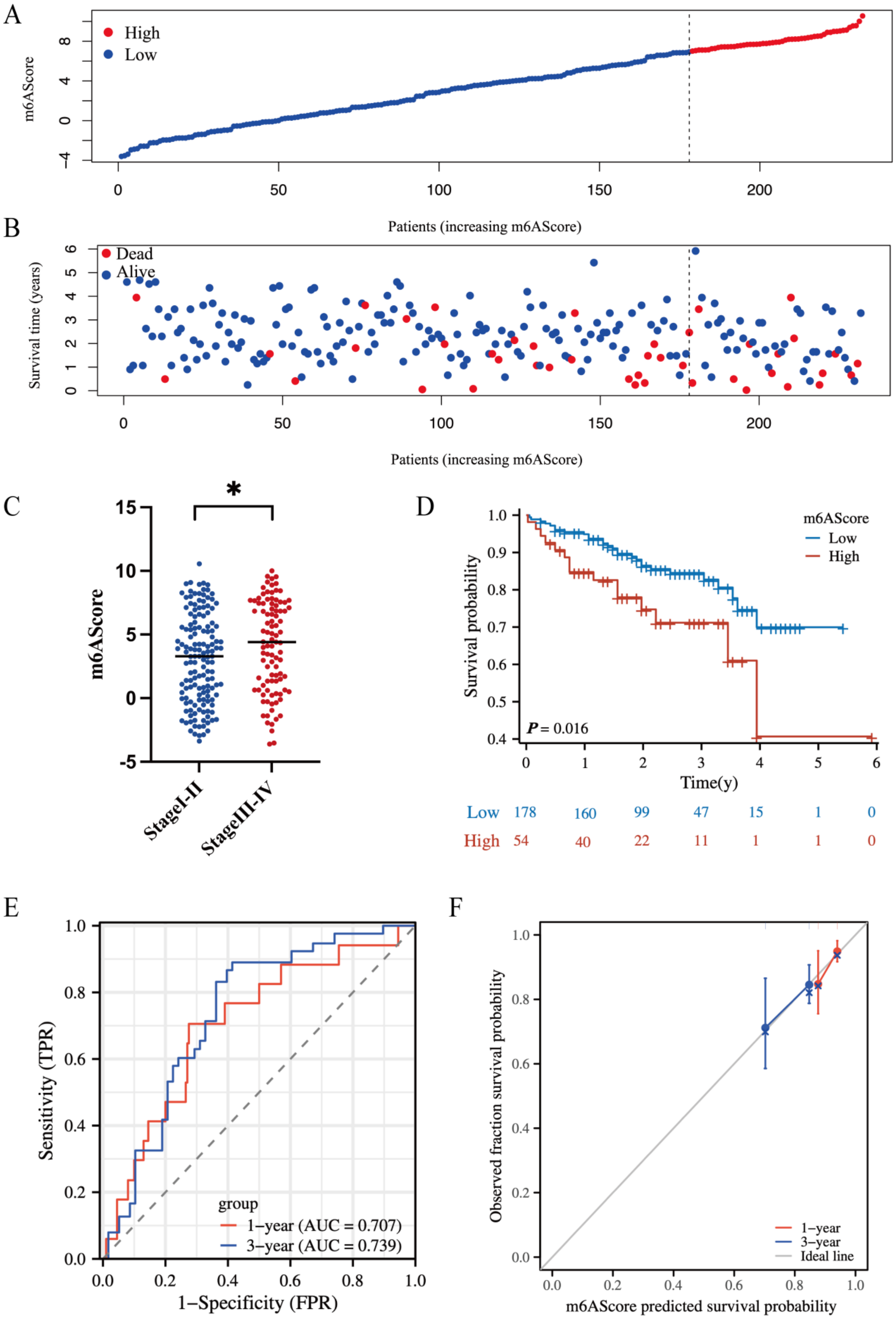
A
High
Low
m6AScore
Patients (increasing m6AScore)
B
Dead
Alive
Survival time (years)
Patients (increasing m6AScore)
C
*
m6AScore
StageI-II
StageIII-IV
D
m6AScore
Low
High
Survival probability
P = 0.016
Time(y)
Low 178 160 99 47 15 1 0
High 54 40 22 11 1 1 0
E
Sensitivity (TPR)
1−Specificity (FPR)
group
1−year (AUC = 0.707)
3−year (AUC = 0.739)
F
Observed fraction survival probability
m6AScore predicted survival probability
1−year
3−year
Ideal line

Figure S4. Validation of the m6A-related gene signature in the ICGC-JP cohort
(A) m6AScore distribution according to the m6A-related signature in independent HCC samples; red represents a high m6AScore (high risk) group and blue represents a low m6AScore (low risk) group. (B) Associations between the m6AScore and survival status; red represents deceased patients and blue represents surviving patients. (C) Box plot showing the relationship between the m6AScore and clinical pathological stage; blue represents stage I–II patients and red represents stage III–IV patients. (D) Kaplan–Meier survival curves showing the low m6AScore group (blue graph) and the high m6AScore group (red graph). (E) Receiver operating characteristics (ROC) curve and area under the ROC of m6AScores for 1 (red graph) and 3 (blue graph) years. (F) Calibration curve of m6AScores for 1 (red graph) and 3 (blue graph) years.
