# Supplementary figures and images for: The m6A-related gene signature stratifies poor prognosis patients and characterizes immunosuppressive microenvironment in hepatocellular carcinoma
Source: Front Immunol. 2023 Aug 25;14:1227593. doi: 10.3389/fimmu.2023.1227593 (PMC10485364; doi:10.3389/fimmu.2023.1227593)

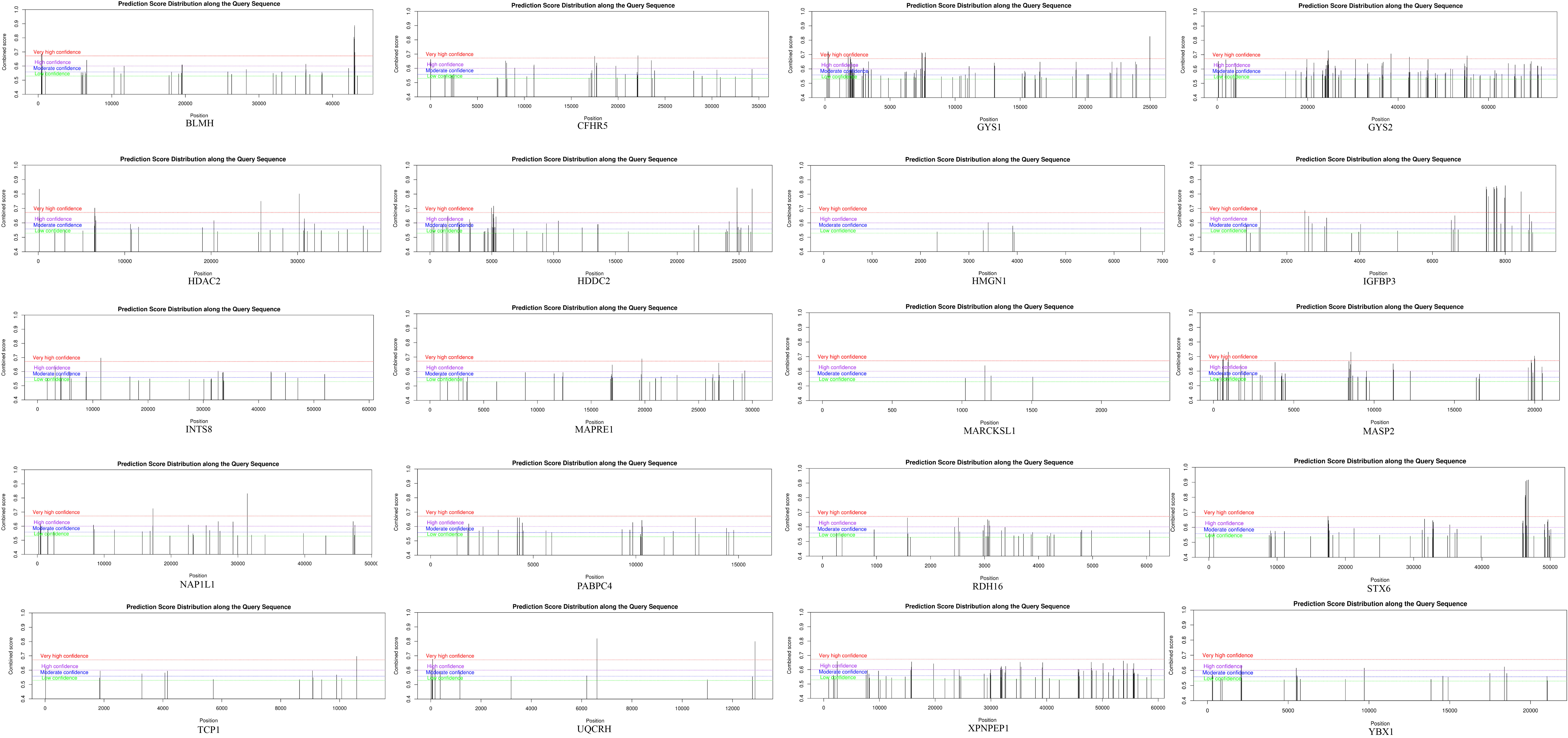

Supplement: Supplementary file 3 [file Image_1.jpeg]
